# The Relationship between Population Structure and Aluminum Tolerance in Cultivated Sorghum

**DOI:** 10.1371/journal.pone.0020830

**Published:** 2011-06-14

**Authors:** Fernanda F. Caniato, Claudia T. Guimarães, Martha Hamblin, Claire Billot, Jean-François Rami, Barbara Hufnagel, Leon V. Kochian, Jiping Liu, Antonio Augusto F. Garcia, C. Tom Hash, Punna Ramu, Sharon Mitchell, Stephen Kresovich, Antônio Carlos Oliveira, Gisela de Avellar, Aluízio Borém, Jean-Christophe Glaszmann, Robert E. Schaffert, Jurandir V. Magalhaes

**Affiliations:** 1 Embrapa Maize and Sorghum, Sete Lagoas, Minas Gerais, Brazil; 2 Institute for Genomic Diversity, Cornell University, Ithaca, New York, United States of America; 3 Centre de Coopération Internationale en Recherche Agronomique pour le Développement (CIRAD), UMR Amélioration Génétique et Adaptation des Plantes méditerranéennes et tropicales, Montpellier, France; 4 Departamento de Biologia Geral, Universidade Federal de Minas Gerais, Belo Horizonte, Brazil; 5 Robert W. Holley Center for Agriculture and Health, U.S. Department of Agriculture – Agricultural Research Service, Cornell University, Ithaca, New York, United States of America; 6 Departamento de Genética, Escola Superior de Agricultura Luiz de Queiroz, Universidade de São Paulo, Piracicaba, Brazil; 7 International Crops Research Institute for the Semi-Arid Tropics (ICRISAT), Patancheru PO, Hyderabad, Andhra Pradesh, Índia; 8 Universidade Federal de Viçosa, Viçosa, Minas Gerais, Brazil; University of Illinois, United States of America

## Abstract

**Background:**

Acid soils comprise up to 50% of the world's arable lands and in these areas aluminum (Al) toxicity impairs root growth, strongly limiting crop yield. Food security is thereby compromised in many developing countries located in tropical and subtropical regions worldwide. In sorghum, *SbMATE*, an Al-activated citrate transporter, underlies the *Alt_SB_* locus on chromosome 3 and confers Al tolerance via Al-activated root citrate release.

**Methodology:**

Population structure was studied in 254 sorghum accessions representative of the diversity present in cultivated sorghums. Al tolerance was assessed as the degree of root growth inhibition in nutrient solution containing Al. A genetic analysis based on markers flanking *Alt_SB_* and *SbMATE* expression was undertaken to assess a possible role for *Alt_SB_* in Al tolerant accessions. In addition, the mode of gene action was estimated concerning the Al tolerance trait. Comparisons between models that include population structure were applied to assess the importance of each subpopulation to Al tolerance.

**Conclusion/Significance:**

Six subpopulations were revealed featuring specific racial and geographic origins. Al tolerance was found to be rather rare and present primarily in guinea and to lesser extent in caudatum subpopulations. *Alt_SB_* was found to play a role in Al tolerance in most of the Al tolerant accessions. A striking variation was observed in the mode of gene action for the Al tolerance trait, which ranged from almost complete recessivity to near complete dominance, with a higher frequency of partially recessive sources of Al tolerance. A possible interpretation of our results concerning the origin and evolution of Al tolerance in cultivated sorghum is discussed. This study demonstrates the importance of deeply exploring the crop diversity reservoir both for a comprehensive view of the dynamics underlying the distribution and function of Al tolerance genes and to design efficient molecular breeding strategies aimed at enhancing Al tolerance.

## Introduction

Aluminum (Al) tolerance has been deemed one of the main breeding targets in acid soil regions [1] and is of particular importance in sorghum in view of its primary role as a staple food and fodder crop in tropical and subtropical African countries [2]. At soil pH values below pH 5.0, rhizotoxic ionic forms of Al are solubilized into the soil solution, damaging sensitive root systems and reducing root growth [3], finally resulting in severe yield losses. Fortunately, genetic variation for Al tolerance can be exploited in breeding programs to improve sustainable production on acid soils. In sorghum, the *Alt_SB_* locus, located on chromosome 3, was first identified as a major determinant for Al tolerance in the sorghum line SC283, explaining 80% of the phenotypic variation in a SC283-derived mapping population [4].

Root organic acid release into the rhizosphere resulting in the formation of stable, non-toxic complexes with Al has long been hypothesized as a major physiological mechanism of tolerance via root Al exclusion in plants [5]. More recently, genes encoding root malate and citrate transporters belonging to the ALMT and MATE families, respectively, have been cloned in wheat (*ALMT1*, [6]), sorghum (*SbMATE*, [7]) and barley (*HvMATE*, [8]). In sorghum, *SbMATE* has been shown to underlie the major Al tolerance locus, *Alt_SB_* [7]. ALMT and/or MATE homologs with a likely role in Al tolerance have also been found in many other species such as maize [9], Arabidopsis [10], [11], wheat [12], rape [13] and rye [14], [15]. Recent studies also indicated the importance of other genes acting both within and outside the organic acid release pathway that influence the ability of plants to deal with Al toxicity. The CH2H2-type zinc finger transcription factor, STOP1 [16], has been shown to regulate both *AtMATE* and *AtALMT* expression [11], [16], and *ART1*, a rice homolog of Arabidopsis *STOP1*, was shown to regulate the expression of several genes with possible roles in rice Al tolerance [17], a response that was also observed for *STOP1* in Arabidopsis [18]. Nevertheless, mechanisms of Al tolerance different than Al-induced organic acid release have been suggested to be mediated by other genes such as those encoding ATP binding cassette (ABC) transporters with a possible involvement in Arabidopsis [19]–[21] as well as in rice Al tolerance [22]. While the specific role of ABC transporters in Al tolerance is yet to be elucidated, these proteins have been hypothesized to mediate Al redistribution from sensitive sites [19], Al sequestration into vacuoles [20] or to promote cell wall modifications [22]. In addition, the rice Nramp metal transporter, *Nrat1*, has recently been proposed to mediate Al uptake as part of a tolerance mechanism based on sequestration of Al away from the cell wall into the symplasm of root cells [23].

*Sorghum* Moench is a heterogeneous genus divided into 5 subgenera within which many species have been described including both rhizomatous and annual types [24]. Accordingly, subgenera *Sorghum* includes *S. halepense* and *S. propinquum*, two rhizomatous species, and *S. bicolor*, which comprises all annual taxa. Three subspecies have been recognized within *S. bicolor* reflecting cultivated taxa, wild types and stabilized weedy derivatives. *Sorghum bicolor* subsp. *bicolor* contains all of the cultivated sorghums [25] that are distributed among 28 grain sorghum types previously classified as species according to the original Snowden system [24]. Cultivated grain sorghums were ultimately classified into five basic botanical races defined based on panicle and spikelet morphological differences [26]. These races are bicolor, caudatum, durra, guinea and kafir, with an additional ten intermediates that were derived from intercrossing among members of the five main races. Sorghum domestication possibly occurred in the northeastern region of Africa at least 5000 years ago [27], giving rise to the early bicolor race [24], [27]. The origin of the guinea race probably took place in tropical West Africa resulting from selection for adaptation to a wet habitat [24], [28]. From this region, the guinea race spread to Malawi and later to southern Africa along the mountains of eastern Africa [24], being subsequently transported to Asia [28]. Guinea sorghums account for more than 70% of the sorghum cultivated in West and Central Africa and may account for more than 50% of all sorghum produced in Africa [29]. The caudatum race probably originated in the area of original domestication of the species [24] from where it spread to West and South Africa. There is evidence suggesting a southern African origin for the kafir sorghums and the origin of the durra race might have taken place in northeastern Africa or Asia [28].

Considering that Al tolerance is a rare event [1], efforts to broaden our still incipient understanding of the diversity of Al tolerance mechanisms in plants parallels the ‘needle in a hay stack’ scenario in germplasm banks [30], where breeders are challenged with skimming through thousands of accessions in search for novel allelic variants for loci underlying desirable traits. To serve as a guide for these efforts, better knowledge on the relationship between population structure and Al tolerance for cultivated sorghums is sorely needed.

Population substructure reflects the evolutionary history of a species [31] and can be understood as the presence of genetically differentiated subgroups in the original population [32]. A myriad of factors can lead to genetic divergence within a population including local adaptation, selection and genetic drift [33], and these factors may result in non-random distribution of important agronomic traits. In cultivated sorghum, genetic diversity patterns are influenced by both racial and geographical origins [34], resulting in well defined subgroups that can be studied for a possible relationship between Al tolerance and population structure.

In the present study, the cultivated sorghum collection described by Deu and colleagues [34] was combined with a sorghum panel that is representative of the lines currently used by the Embrapa acid soil breeding program [35]. The combined cultivated sorghum panel was then subjected to a population structure analysis. Our analysis strongly indicates that Al tolerance is a rare trait that is not randomly distributed considering the diversity patterns observed in cultivated sorghums. In addition, a wide range of diversity was observed for dominance behavior related to the Al tolerance trait, which ranged from almost complete recessivity to almost complete dominance. Finally, our population structure analysis allowed us to make inferences with regards to the origin and evolution of Al tolerance mutations in light of the domestication history leading to cultivated sorghums.

## Results

### Al tolerance variation

At {27} µM Al^3+^ (Table S1), 80% of the sorghum accessions were sensitive to Al (*RNRG*_5d_<30%), 14% were intermediately tolerant (30%<*RNRG*_5d_<80%), and only 6% or 16 sorghum accessions showed *RNRG*_5d_>80%, thus being classified as highly Al tolerant (only these lines were designated Al tolerant in this paper). Accounting for lines that are breeding derivatives from known Al tolerant sources (e.g. the sorghum line 9929034 is derived from SC566, and CMS226 and CMS227 are derived from SC283), only 5% of the whole panel were found to be highly tolerant to Al. Sorghum Al tolerance is inducible over time, significantly increasing after two to three days of Al exposure [7]. Here we have used an *I*nduction of *R*oot *G*rowth (*IRG*) index which is generated by dividing the daily rate of root growth calculated between the 3^rd^ and 5^th^ days of Al exposure by that obtained between the 1^rst^ and the 3^rd^ days. Differences in magnitude of the induction response were also observed across the panel with most of the accessions showing root growth inhibition at varying degrees (*IRG*<1), and only 26 accessions showing induction of root growth for 3 to 5 days of Al exposure compared to root growth for 1 to 3 days in Al (*IRG*>1). The induction response varied substantially among these 26 accessions, from nearly 1 (i.e. almost constant growth rates) to a 100% increase in the rate of root growth between days three and five in Al.

At {39} and {60} µM Al^3+^, the sorghum accession IS14351 did not group with any other accession, showing the highest relative net root growth, which indicates it is the most Al tolerant accession in the panel.

**Figure 1:**
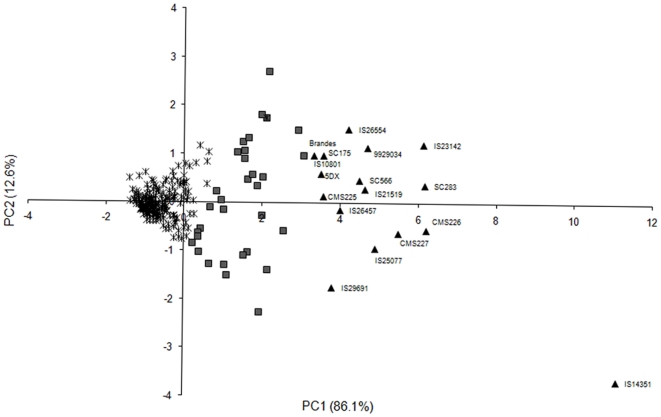
Accession Scores for Principal Components (PC) 1 and 2. PC analysis was undertaken with Relative Net Root Growth at 3 (*RNRG*_3d_) and 5 (*RNRG*_5d_) days of Al exposure and Induction of Root Growth (*IRG*). The cumulative proportion of the total variance that is explained by each PC (%) is shown. Triangles represent Al tolerant accessions (*RNRG*_5d_>80%), squares represent intermediate accessions (30%<*RNRG*_5d_<80%) and asterisks represent Al sensitive accessions (*RNRG*_5d_<30%).

Principal Component Analysis allowed us to identify the first and second principal components as responsible for 98.7% of the total Al tolerance variation (Figure 1 and Table S2). The first principal component (PC1), whose linear combination has positive eigenvector coefficients for all variables, can thus be interpreted as a general Al tolerance index, whereas the second principal component (PC2), explaining 12% of the variation, contrasts relative root growth assessed at 3 and 5 days of Al exposure to the induction response (Table S2). The majority of the sorghum accessions in the diversity panel showed low scores for both PC1 and PC2 (Figure 1), reflecting the high frequency of Al sensitive accessions in the diversity panel. A significant spread in PC2 scores was observed with increasing tolerance (PC1>0), with maximum amplitude being reached at PC1 near 2. Highly Al tolerant accessions (PC1>3.5) showed PC2 scores in general between approximately +1.5 (IS26554) and −1.7 (IS29691). The relative importance of the induction response to *RNRG* varied, being substantial for accessions such as IS26554, similar in IS26457/CMS225 and smaller in IS29691 (Figure 1). The highly Al tolerant accession, IS14351, showed the lowest PC2 score, indicating a relatively lower importance for the induction response in this highly Al tolerant line.

### Genetic and expression analysis of Al tolerance

**Figure 2:**
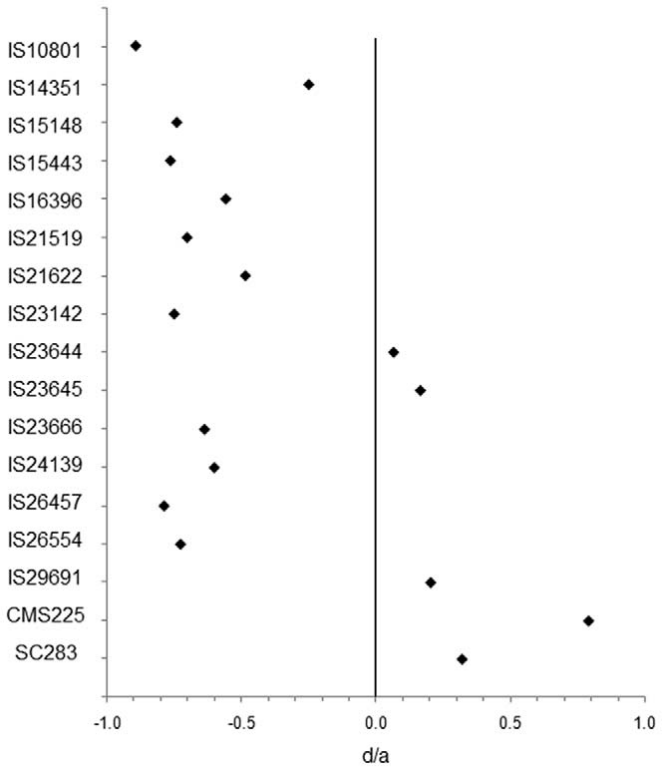
Gene action estimate for Al tolerance. The degree of dominance is shown as the ratio between dominance (d) and additive (a) effects. Totally additive (d/a = 0) gene action is depicted by a continuous line drawn vertically to the x-axis. As a convention, Al tolerance was considered a recessive trait when d/a≤−0.7, partially recessive for −0.7<d/a<−0.3, additive for −0.3≤d/a≤+0.3, partially dominant for +0.3<d/a<+0.7 and dominant when d/a≥+0.7. Further information concerning this dataset is shown in Table S4.

We undertook linkage analysis between Al tolerance and markers flanking *Alt_SB_* to assess the role of the Al tolerance locus in the donor accessions. Because 80% of the accessions in the panel were Al sensitive, linkage analysis focused primarily on populations derived from the Al tolerant accessions. Populations derived from two intermediate accessions, IS21849 and IS23645, were also included in this analysis. Linkage analysis revealed that Al tolerance in IS14351, IS21519, IS21849, IS23645 and IS26554 can be attributed to the *Alt_SB_* locus, whereas significant marker-trait associations were not found for BC families derived from IS23142, IS26457 and IS29691 (Table S3). However, analysis of Al tolerance for parents and derived F_1_ hybrids indicated additive gene action (−0.3≤d/a≤+0.3) for 4 Al tolerance donors, whereas Al tolerance in 11 out of the 17 sorghum accessions was either a recessive (d/a≤−0.7) or partially recessive trait (−0.7<d/a<−0.3) (Figure 2 and Table S4). The sorghum accessions, CMS225 and SC283, showed the highest degree of dominance and strict complete dominance (d/a = 1) was never observed in this study. It should be noted that the power to detect genetic linkage in a backcross population decreases as gene action approaches complete recessivity, although this extreme situation was never observed in our dataset. Considering the rather recessive behavior of Al tolerance in IS23142 and IS26457, even with the lack of linkage with markers flanking *Alt_SB_*, we cannot rule out the possibility that Al tolerance in these accessions is due to partially recessive *Alt_SB_* alleles. However, this possibility seems less likely for IS29691 in view of its rather additive mode of gene action for Al tolerance.

**Figure 3:**
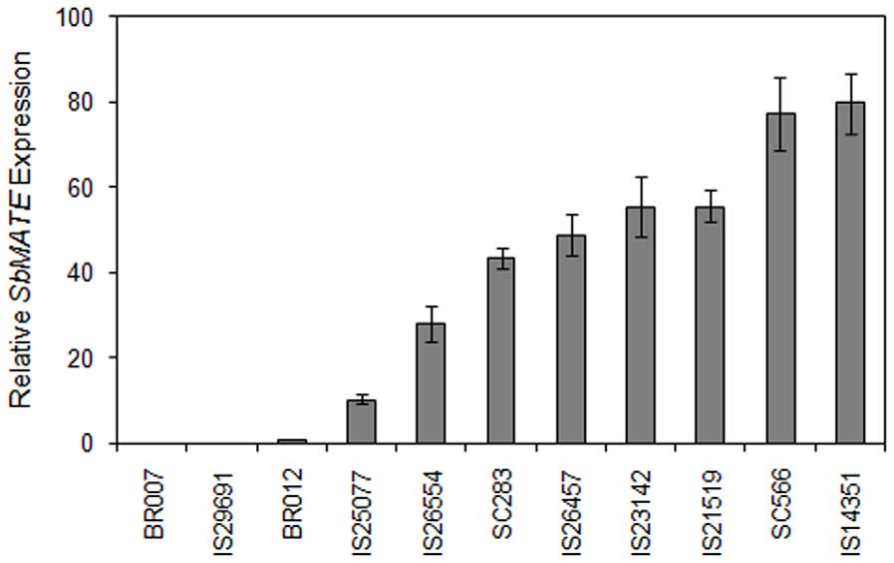
Expression analysis of *SbMATE*. *SbMATE* relative expression was determined using quantitative real-time PCR with expression in the Al sensitive line, BR012, as a reference. 18S ribosomal RNA was used as internal control. The first centimeter from root apices cut from roots of intact plants exposed to {27} µM Al^3+^ in nutrient solution at pH 4.0 for 5 days were harvested for total RNA isolation. Twenty-eight apices per experimental unit (genotype) were collected and the bars indicate standard deviations based on 3 technical reps.

We then studied expression for *SbMATE*, which underlies *Alt_SB_*, in 7 Al tolerant accessions including IS14351, IS21519 and IS26554, whose Al tolerance was found to be due to *Alt_SB_* according to our genetic analysis, and IS23142, IS29691 and IS26457, for which non-significant marker-trait associations were observed (Table S3). Two known sources of Al tolerance due to *Alt_SB_*, SC283 and SC566, in addition to the Al sensitive standards, BR007 and BR012 (Table S1 and [35]), were also included as controls. All Al tolerant accessions except for IS29691 exhibited *SbMATE* expression levels significantly higher than that in the Al sensitive standards, BR012 and BR007 (Figure 3). Expression in the tolerant lines ranged from ∼10- to ∼80-fold higher than that observed in BR012. This is consistent with a strong role of *Alt_SB_* in Al tolerance for these accessions despite the recessive mode of gene action observed in some sources. In agreement with our expectations, due to its extremely low level of *SbMATE* expression, only IS29691 is likely to strongly rely on Al tolerant loci distinct from *Alt_SB_*.

### Distribution of Al tolerance with respect to racial classification

**Figure 4:**
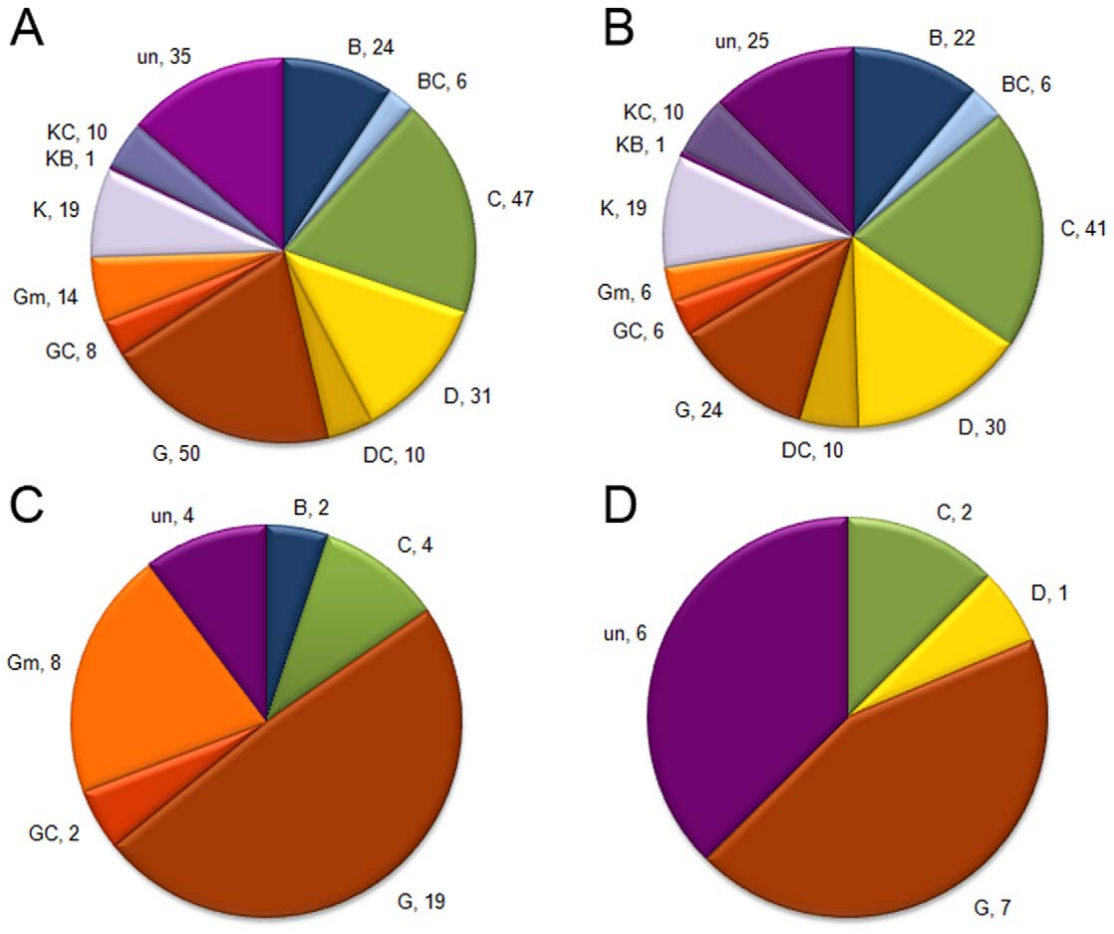
Racial distribution with respect to the Al tolerance phenotype. (A) whole diversity panel; (B) Al sensitive accessions (*RNRG*_5d_<30%); (C) Al intermediate accessions (30%<*RNRG*_5d_<80%); (D) Al tolerant accessions (*RNRG*_5d_>80%). B: bicolor; C: caudatum; D: durra; G: guinea; Gm: guinea margaritiferum; K: kafir; DC: intermediate durra-caudatum; BC: intermediate bicolor-caudatum; GC: intermediate guinea-caudatum; KC: intermediate kafir-caudatum; KB: intermediate kafir-bicolor; un, unknown. Numbers after commas indicate the number of accessions within each racial class.

The five basic morphological races were represented in the diversity panel with a larger and similar representation for the guinea and caudatum races (Figure 4A). Aluminum sensitive accessions tended to be randomly distributed across the major sorghum races with a slightly higher frequency in caudatum sorghums (Figure 4B). Nonetheless, the racial distribution for intermediate and Al tolerant accessions was strikingly different. The vast majority of intermediate accessions, 19, were found to be members of the guinea race with an additional 8 and 4 accessions belonging to guinea margaritiferums and the caudatum races, respectively. The remaining intermediate accessions were evenly distributed at a lower frequency within guinea-caudatums, bicolors or were uncharacterized for racial origin (Figure 4C). Seven of the sixteen Al tolerant accessions were guinea sorghums and two were caudatums, with one accession, IS23142, morphologically classified as a durra type. The six remaining Al tolerant accessions were breeding derivatives (Figure 4D). Because the sorghum panel is unbalanced with respect to racial representation, we undertook a Chi-square test for independence based on a six (number of accessions in each of the five basic sorghum races and guinea margaritferum)×2 (Al tolerant + intermediate and Al sensitive) contingency table. The results (χ^2^ = 50.8, P[χ^2^>50.8] = 1.3E-14) indicated that the distribution of Al tolerance cannot be explained solely by differences in racial representation in the diversity panel, thus significantly departing from a random pattern.

**Figure 5:**
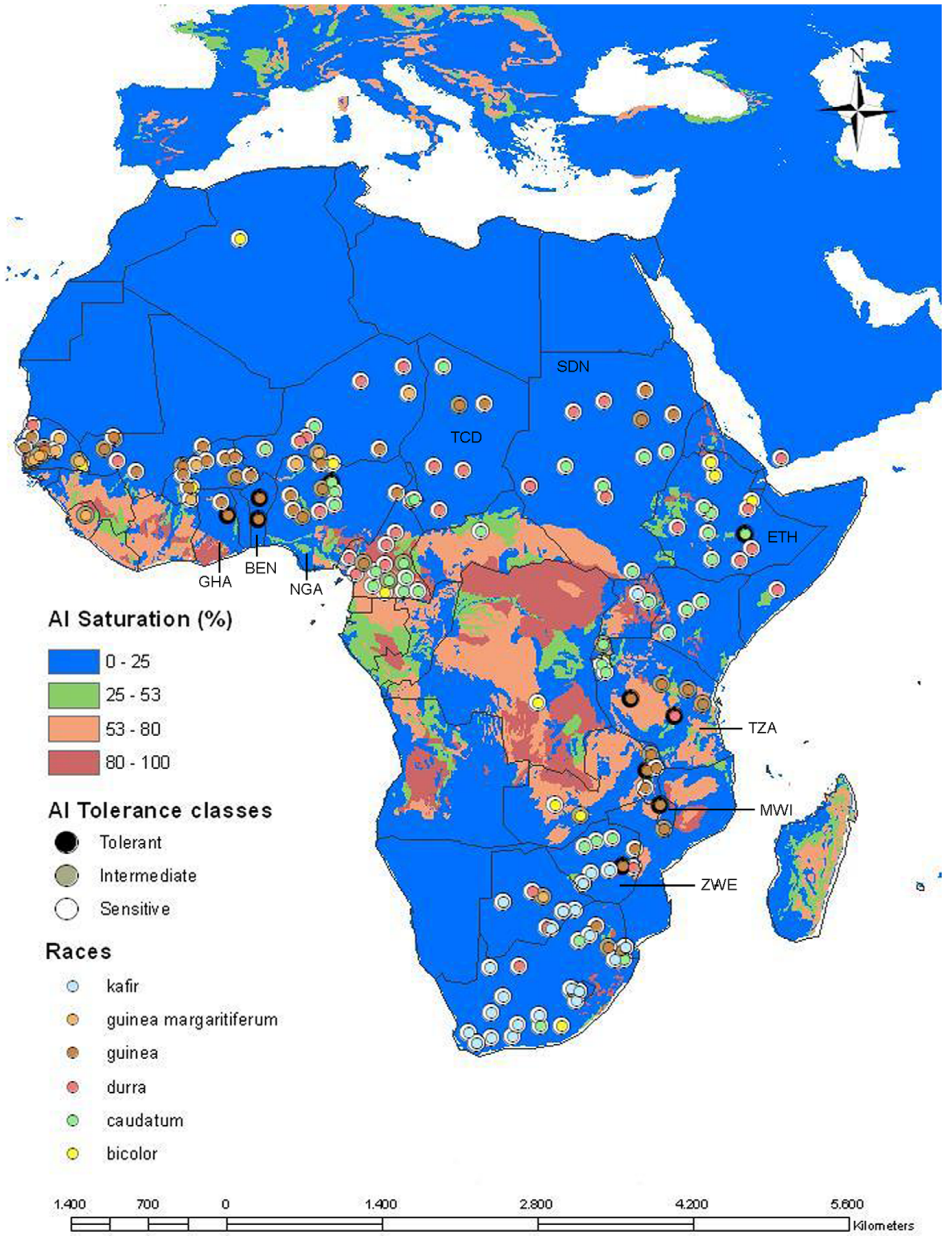
Geographical and racial distribution of the sorghum accessions and Al toxicity in Africa. Accessions were plotted on the map based on the latitude and longitude coordinates found in http://www.icrisat.org/sorghum/Project1/pfirst.asp when available. Accessions lacking those coordinates were plotted randomly within the known country of origin. Outer circles indicate the classes of Al tolerance whereas inner circles indicate racial classification [26]. Racial classifications can be found in [34] and http://www.ars-grin.gov. The soil data set is based on the Fertility Capability Classification (FCC, [60]). Country abbreviations are shown for countries cited in the text as well as those where Al tolerant accessions occur: Ghana (GHA), Benin (BEN), Nigeria (NGA), Chad (TCD), Sudan (SDN), Ethiopia (ETH), Tanzania (TZA), Malawi (MWI) and Zimbabwe (ZWE).

Sorghum accessions in the diversity panel were then geographically represented on a soil map of Africa depicting the distribution of Al saturation classes (Figure 5). Except for two accessions coming from Sudan and Chad, intermediate accessions appeared to be more frequent in West and East Africa. The distribution of Al tolerant accessions coincided in general with the distribution of the intermediate accessions, but the former were geographically more tightly clustered in West Africa compared to a broader distribution in East Africa, across Ethiopia and Tanzania and South/East Africa, in Malawi and Zimbabwe. At the level of resolution of the soil map, Al tolerant accessions from South/East Africa appear to be originated in areas particularly prone to Al toxicity, in soils with Al saturation above 25%.

### Analysis of population structure based on SSR markers

A total of 501 alleles were revealed by 38 SSR loci genotyped in 254 sorghum accessions. Within those, 399 showed minor allele frequencies under 10%. The average number of alleles per locus was 13.2, ranging from 2 for the marker locus, *Xtxp136*, to 29 for *Xgap206*. The Polymorphic Index Content (PIC) value over the 38 SSR markers averaged 0.65, ranging from 0.19 for marker *mSbCIR246* to 0.93 for markers *Xgap206* and *Xtxp321* (Table S5).

Upon population structure analysis, the Ln(k) vs. k curve showed a steep increment in model likelihood up to k = 4 although additional but apparently slighter increments occurred between 5 and 12 subpopulations (Figure S1). Using the Δk criterion, the most evident level of differentiation was observed with k = 4, but additional peaks, although much less evident, were also detected at k = 6 and k = 12 (Figure S2). Nevertheless, the largest proportion of individuals assigned to a specific cluster with a cluster membership probability higher than 0.8 was obtained with k = 4 and k = 6, with 81 and 71%, respectively, contrasting with ∼60% for k = 12. However, particularly in sorghum, where hybridization between sorghum races is a common event, cluster membership should not be adopted as the sole criterion to define the most likely number of subpopulations. Thus, we subsequently analyzed in detail the nature of the clusters obtained setting k at 4 and 6 subpopulations. The corresponding clusters for k = 4 (Figure S3A) were composed of guinea accessions from western Africa and guinea margaritiferum (k4Q1), durra accessions from central-eastern Africa and from Asia, bicolor and caudatum accessions from Asia (k4Q2), caudatum accessions from Africa, a group of transplanted caudatum and durra accessions from the Lake Chad region, and lines from the Embrapa collection and the US (k4Q3), and kafir and guinea accessions from southern Africa (k4Q4). At k = 6, the former k4Q3 group was separated into k6Q2, which included caudatum accessions from Africa and a group of transplanted caudatum and durra accessions from the Lake Chad region, and k6Q3, with lines from the Embrapa collection and the US. In addition, the k4Q4 group was separated into k6Q4, which included kafir accessions from southern Africa and k6Q6, which included guinea accessions from southern Africa (Figure S3B, Table S6). Based on these results, we believe that six subpopulations result in a meaningful representation of the genetic diversity patterns underlying this panel, which led us to define k = 6 as the starting point to look into the distribution of Al tolerance in sorghum.

### Population structure and Al tolerance in sorghum

**Figure 6:**
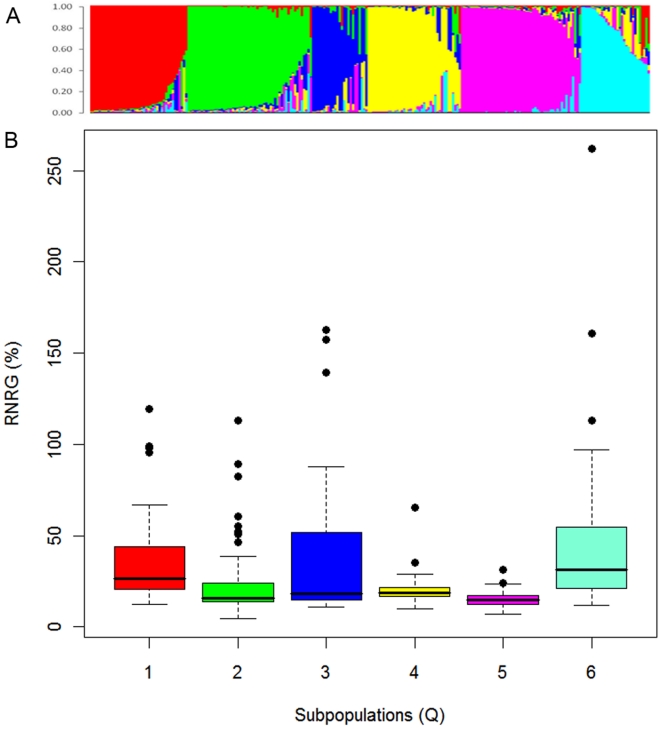
Organization of individual sorghum accessions into six sorghum subpopulations and distribution of Al tolerance. Subpopulation assignments (A) and (B) box plots for Relative Net Root Growth (*RNRG*_5d_) for each subpopulation: Q1 (guinea accessions from western Africa and guinea margaritiferum accessions), Q2 (caudatum accessions from Africa and group of transplanted caudatum and durra accessions from Lake Chad region), Q3 (lines from the Embrapa collection and US), Q4 (kafir accessions from southern Africa), Q5 (durra accessions from central eastern Africa and from Asia; bicolor and caudatum accessions from Asia), Q6 (guinea accessions from southern Africa and Asia).

The distribution of Al tolerance within each of the six subpopulations defined with STRUCTURE (Figure 6A) is shown in Figure 6B. The distributions were in general asymmetric and skewed towards Al sensitivity (*RNRG*_5d_<∼50%). Intermediate accessions were predominantly clustered in Q1 (guinea accessions from western Africa and guinea margaritiferum accessions), Q3 (lines from the Embrapa collection and US) and Q6 (guinea accessions from southern Africa and Asia), resulting in greater interquartile range for Q1, Q3 and Q6 compared to the other subpopulations. Al sensitive accessions were mainly clustered in Q2 (caudatum accessions from Africa and the group of transplanted caudatum and durra accessions from Lake Chad region), Q4 (kafir accessions from southern Africa) and Q5 (durra, bicolor and caudatum accessions from eastern Africa and Asia). Al tolerant accessions appear as outliers in Figure 6B and were again predominantly present in Q1, Q3 and Q6 but were also present in Q2 (caudatum types).

Due to different population sizes and unequal variances within subpopulations for Al tolerance traits, the Kruskal-Wallis test was applied as suggested by Lin and collaborators [36], confirming that there are differences among subpopulations for all traits related to Al tolerance. The non-parametric *lsd* test indicated subpopulations Q1, Q3 and Q6 to be in general superior in terms of Al tolerance traits (Table 1).

**Table 1:** Statistical analysis for Al tolerance traits in six subpopulations (Q).

Q	N	*RNRG*_3d_						*RNRG*_5d_					*VRD*					*IRG*			
Q1	47	117.7		b	c	d		179.3	a				176.8	a				171.3	a		
Q2	58	103.9		b	c	d	e	106.6		b	c	d	132.1		b	c		120.9		b	c
Q3	23	144.3	a	b				134.3	a	b			153.8	a				117.4		b	c
Q4	43	119.4		b	c			124.2		b	c		99.8		b	c	d	132.4	a	b	
Q5	54	71.6					e	72.2				d	81.8				d	86.7			c
Q6	29	196.1	a					188.0	a				143.8	a	b			146.1	a	b	

Q1, guinea accessions from western Africa and guinea margaritiferum; Q2, caudatum accessions from Africa and group of transplanted caudatum and durra accessions from Lake Chad region; Q3, Lines from Embrapa collection and US; Q4, kafir accessions from southern Africa; Q5, durra, bicolor and caudatum accessions from eastern Africa and Asia; Q6, guinea accessions from southern Africa and Asia.

Statistical analysis for Relative Net Root Growth (*RNRG*) after 3 and 5 days of Al exposure, Visual Root Damage (*VRD*) and Induction of Root Growth (*IRG*) in six sorghum subpopulations. Means followed by the same lower-case letters are not statistically different by the non-parametric least significant difference (*lsd)* test (P<0.05). N: Number of individuals within each subpopulation.

**Figure 7:**
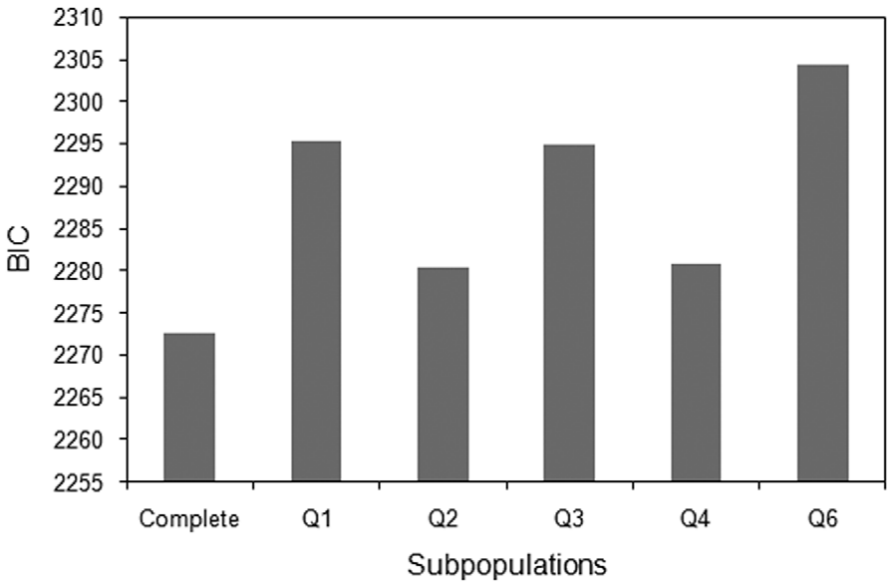
Model comparisons for Al tolerance. Bayesian information criterion (BIC) for the complete Q+K model (complete) and for reduced models sequentially excluding subpopulations Q1, Q2, Q3, Q4 and Q6 (excluded subpopulations are shown in the x-axis). The subpopulation Q5, which contained only Al sensitive accessions, was excluded in all cases.

Finally, we undertook a series of model selection steps as an attempt to more formally isolate the individual contribution of the different subpopulations to Al tolerance. Our rationale is based on the idea that removing a subpopulation that captures a significant proportion of the Al tolerance variation from the model should result in a decrease in model likelihood. The complete model showed the lowest Bayesian Information Criterion (BIC) value, indicating that all subpopulations are important to explain the observed variation in Al tolerance (Figure 7). However, excluding subpopulations, Q1, Q3 and Q6 resulted in a stronger reduction in model performance when compared to excluding the remaining subpopulations. Moreover, based on the increment in BIC estimates, guineas from southern Africa and Asia (Q6) appear to be the most important in capturing Al tolerance variation, followed by guinea accessions from western Africa and guinea margaritiferum (Q1), and lines from the Embrapa collection and the US (Q3). The contribution of the remaining subpopulations, Q2 and Q4, although significant, was lower than that of Q6, Q1 and Q3. This is expected, considering the lower representation of intermediate accessions in Q2 and Q4, whose removal caused a nearly equal increase in BIC estimates. We also used the PROC STEPWISE procedure implemented in SAS with the MAXR option to obtain the proportion of the variance explained by population structure alone, which was approximately 16%. This indicates that although the incorporation of population structure covariates is important to control for false positives in association analysis for Al tolerance, a substantial fraction of the phenotypic variance should still persist and can be potentially assigned to Al tolerance QTL.

## Discussion

The sorghum panel used in this study was assembled to represent the genetic diversity present in cultivated sorghums [34], thus allowing us to look in detail into a possible relationship between population structure and Al tolerance with a focus on sorghum production on acid soils. A similar low frequency for Al tolerance was also observed by Reddy and collaborators [37] based on field screening and is consistent with Al tolerance being a derived state [1], with a possible relatively recent origin of Al tolerance mutations in sorghum.

Our previous [35] and present genetic analyses indicate that 11 out of the 16 highly Al tolerant accessions rely on the *Alt_SB_* locus to express their tolerance. Because of the rather recessive nature of some *Alt_SB_* alleles that could lead to false negatives in the genetic analysis, we also studied expression for *SbMATE*, which underlies *Alt_SB_*, in a subset including 4 of the 5 remaining Al tolerant accessions in addition to other Al tolerant accessions for which our genetic analysis confirmed a role for *Alt_SB_* in Al tolerance control. All Al tolerant accessions except for one showed expression levels significantly higher than that in the Al sensitive standards, BR007 and BR012. Considering that Al tolerance and *SbMATE* expression are highly correlated [7], our data supports a role for *Alt_SB_* in providing tolerance to the vast majority of the Al tolerant accessions in the diversity panel. In addition, tolerant lines were found to vary for *SbMATE* expression levels, suggesting contrasting allelic effects. This expands on our previous findings indicating substantial diversity for Al tolerance control at the *Alt_SB_* locus, which in a small, 12-member panel, was largely due to an allelic series at *Alt_SB_* encoding highly variable Al tolerance phenotypes [35].

Population structure analysis revealed that Al tolerance is by no means randomly distributed across the diversity continuum but instead is rather specific to certain genetically differentiated subgroups featuring specific racial and geographical origins. Particularly, the guinea subpopulations Q1 and Q6 are important repositories of Al tolerance in sorghum. Although the caudatum subpopulation, Q2, appears to be relatively less important than Q1, Q3 and Q6 in explaining the variation for Al tolerance in the panel according to our Q+K model, this subpopulation included eight lines with *RNRG* values between 40 and 100. This indicates that sorghum subpopulations containing caudatum types may also be useful for the identification of Al tolerance donors. Interestingly, the only non-guinea/caudatum accession that was found to be Al tolerant, IS23142, morphologically classified as a durra type, showed high membership coefficients to guinea subpopulations Q1 and Q6, suggesting a guinea-durra transfer of Al tolerance. The high level of tolerance observed within Q3, a subpopulation with a predominance of lines from the Embrapa collection and the US, reflects the fact that some of those lines have been purposely selected for breeding Al tolerant sorghums for the Brazilian acid soils, also reflecting the presence of Al tolerant breeding derivatives [35].

Correlation between population structure and variation for a phenotypic trait has been reported and may result from adaptation and/or genetic drift [38], [39]. In maize, a deletion allele of the *D8idp* gene, which is associated with flowering time, was found in high frequency among Northern Flint material and in low frequency among tropical material, likely resulting from diversifying selection for flowering time [39]. Interestingly, the guinea race is the main race of sorghum cultivated in West Africa due to its adaptation to a range of stresses commonly found there, including poor soil fertility and low soil pH [40]. This suggests that the strong relationship between population structure and Al tolerance in sorghum is not solely caused by genetic drift and may be the result of local adaptation to acid, Al toxic soils. Considering that those soils can be distributed in rather localized regions, thus escaping the resolution level of our soil map, a more detailed soil characterization in West Africa with regards to Al toxicity is needed to gain additional insights into this hypothesis. The local adaptation hypothesis is reinforced by the fact that Al toxicity has indeed been documented to impair sorghum production in West Africa [41], [42].

The fact that the vast majority of the Al tolerant accessions in the diversity panel were either guinea types or were genetically closely related to guinea sorghums from West and South/East Africa, leads us to speculate that Al tolerance mutations were originated after the initial migration from the original area of sorghum domestication between Sudan and Ethiopia [2], [27], arising in West Africa after the guinea race differentiated from the primordial bicolor types. Supportive of this hypothesis is the presence of Al tolerant accessions in the Malawi region, which is thought to be a secondary center where guinea sorghums occur [24], [26], [43]. Interestingly, one of the most Al tolerant accessions in the diversity panel, SC566, a caudatum type from Nigeria, clustered with guinea sorghums, reinforcing the possibility for a single racial origin with subsequent interracial spread of Al tolerance genes in sorghum. In fact, it is known that the guinea race is sympatric with all four of the other basic races of sorghum and interracial hybrids among them are occasionally observed, which are commonly encountered in drier areas from Nigeria to Uganda [28].

The accessions in the diversity panel showed strikingly different modes of gene action for Al tolerance, with the vast majority showing recessive gene action at varying degrees. Interestingly, the dominance level in newly adaptive genes for insecticide resistance has been shown to be extremely plastic, varying from almost recessive to almost complete dominance [44]. Considering the plausible possibility that the rather new Al tolerance mutations were originally recessive in nature, we are then presented with the question of how dominance arose for Al tolerance genes. Although it has been the subject of great debate (reviewed by Bourget [45]), the hypothesis of dominance arising from evolutionary change has been proposed [46]. More recently, the degree of dominance for QTL controlling differences in plant and influorescence architecture between maize and teosinte was found to be greater in the maize background [47]. This observation led the authors to hypothesize that changes in gene action could possibly result from selection during the domestication process for modifier loci that enhance the expression of the trait in the heterozygote. The strong correlation between Al tolerance and *SbMATE* expression and the highly monomorphic nature of the *SbMATE* coding region suggest an important role for regulatory polymorphisms in Al tolerance controlled by the *Alt_SB_* locus [7]. Along those lines, the fact that MATE genes have been found to be modulated by transcription factors such as STOP1 [11] leads us to raise the hypothesis that dominance in the case of Al tolerance is an acquired state, with a possible origin at modifier loci interacting with Al tolerance genes. One possible precedent for this is the acethylcholinesterase gene conferring insecticide resistance, which showed extremely plastic dominance behavior [48]. Clearly, in the case of Al tolerance, a more specific study on background effects modulating the expression of Al tolerance genes is needed to gain further insights into this hypothesis. In addition, our experimental design allowed us to assess the degree of dominance related to the Al tolerance trait as a whole, whereas the dominance behavior for *Alt_SB_* was not individualized. Although our data strongly suggest a pivotal role for *Alt_SB_* in conferring Al tolerance, evidence for other Al tolerance genes has been found both here for IS29691 and in our previous studies [35].

The strong relationship observed in the present study between Al tolerance and population structure and the significant plasticity in dominance behavior indicate that the dynamics involving the distribution and function of major Al tolerance genes is much more complex than initially suggested by simple inheritance outcomes in the pioneering genetic studies with a few parental genotypes. A more comprehensive and detailed view of plant Al tolerance enabling powerful molecular breeding strategies will require a detailed understanding of the evolutionary history leading to Al tolerance loci in each species.

## Materials and Methods

### Genetic stocks

Two-hundred and nine accessions from the landrace collection described in [34] and forty-five inbred lines that are frequently used in breeding programs in the US and Brazil formed a combined panel that was used in this study.

Seventeen F_1_ hybrids were generated by crossing different accessions, which ranged from intermediate to high Al tolerance, to the Al sensitive line BR007, to investigate the mode of gene action for Al tolerance. For a genetic analysis of Al tolerance based on the *Alt_SB_* locus, 8 F_1_ hybrids derived from 2 moderately and 6 highly Al tolerant accessions were backcrossed to BR007 to generate backcross one F_1_ (BC_1_F_1_) populations.

### Assessment of Al tolerance in nutrient solution

Analysis of Al tolerance was conducted in nutrient solutions containing either 0 or 148 µM Al, which correspond to free Al^3+^ activities of {0} and {27}µM Al^3+^ (values inside brackets indicate Al^3+^ activity estimated with the speciation software, GEOCHEM-PC [49]). A subset of 27 accessions including all Al tolerant accessions determined at {27}µM Al^3+^ were re-screened at 0, 222 and 360 µM Al, which correspond to free Al^3+^ activities of {0}, {39} and {60} µM Al^3+^. The highly Al tolerant line, SC566, and the Al sensitive line, BR007, which had been previously identified as such by [35], were included as controls. The experiments consisted of a completely randomized design with two replications and seven plants per replication. Hydroponic analyses of Al tolerance were undertaken as described in [35]. Briefly, seeds of each genotype were germinated for four days and seedlings were transferred to containers with nutrient solution lacking Al (pH 4.0) placed in a growth chamber with 27°C day and 20°C night temperatures, a light intensity of 330 µmol photons m^−2^ s^−1^ and a 12-h photoperiod. After 24 h of acclimation, the *i*nitial *l*ength of each seedling's root growing in *c*ontrol solution (*ilc*) was measured. The solution was then replaced by nutrient solution of identical composition but containing either no Al or {27}, {39} or {60} µM Al^3+^ supplied as AlK(SO_4_)_2_.12H_2_O. *F*inal root *l*engths under *Al* treatment (*flAl*) or *c*ontrol solution (*flc*) were obtained after three and five days of exposure to Al. For each inbred line, mean values of *r*elative percent *n*et *r*oot *g*rowth (*RNRG*_3d_ and *RNRG*_5d_, where d is the Al exposure period in days) at each Al activity of {27}, {39} and {60} µM Al^3+^, were estimated by dividing the net root growth under Al treatment (*flAl−ilc*) by the net root growth without Al (*flc−ilc*).

For the genetic analysis of Al tolerance control at *Alt_SB_*, 8 BC_1_F_1_ families were phenotyped for Al tolerance at {27} µM Al^3+^. An independent control lacking Al cannot be employed in families segregating for Al tolerance due to the genetically dissimilar nature of individual plants. Thus, Al tolerance was assessed on an individual plant basis as described in detail in [35], by estimating the degree of root growth inhibition caused by Al over a five-day exposure period relative to the control root growth. *R*elative *r*oot *g*rowth (%) was calculated with the formula *RRG* = [(*flAl*−*flc*)_5d_/((*flc−ilc*)_1d_×5)]×100 where d is the Al exposure period measured in days.

To investigate the mode of gene action for Al tolerance, 17 F_1_ hybrids having BR007 as the common Al sensitive parent were evaluated at {27} µM Al^3+^, including the parents of each cross as controls, and RRG was estimated. The experiments consisted of completely randomized designs with at least 7 plants per genotype.

The sorghum accessions were also inspected for root damage after five days of Al exposure and a *V*isual *R*oot *D*amage (*VRD*) scale ranging from 1 (root apices heavily damaged) to 5 (root apices undamaged) was applied. Three independent evaluations were carried out for estimating *VRD* means.

Al tolerance in sorghum has been reported to be inducible over time, significantly increasing after two to three days of Al exposure [7]. In the current study, an *I*nduction of *R*oot *G*rowth (*IRG*) index was estimated by dividing the daily rate of root growth calculated between the 3^rd^ and 5^th^ days of Al exposure by that obtained between the 1^rst^ and the 3^rd^ days. *IRG* values less than one indicate that the rates of root growth recorded between days 3 and 5 of Al exposure were smaller than those between days 1 and 3, values equal to one indicate constant root growth rates whereas induction of root growth results in *IRG*>1, reflecting higher rates of root growth between days 3 and 5 relative to those between days 1 and 3 of Al exposure.

### Analysis of *SbMATE* expression via Quantitative Real-Time Reverse Transcription (RT) PCR

Sorghum seedlings were grown following the same methods used for assessment of Al tolerance in nutrient solution containing {27} µM Al^3+^ in a growth chamber under controlled environmental conditions. Each experimental unit (genotype) consisted of the first centimeter of root apices collected from 28 intact plants, 5 days after Al treatment imposition. These 28 plants per genotype were divided in 4 sets (7 plants per set) and each set was randomized inside the growth chamber.

Total RNA was extracted from tissue samples using the RNeasy Plant Mini Kit (Qiagen, Valencia, CA) and 10 U of DNase I (RNase free) from the same manufacturer were added to each sample following incubation at room temperature for 15 min. First-strand cDNA was synthesized using 2 µg of total RNA with the High-Capacity cDNA Reverse Transcription Kit (Applied Biosystems, Foster City, CA).

*SbMATE* transcripts were quantified with using the TaqMan Gene Expression kit on the ABI Prism 7500 Real Time PCR System (Applied Biosystems, Foster City, CA). A series of cDNA dilutions were used for making standard curves both for *SbMATE* transcripts and for 18S RNA which was used as the internal reference. Then, the selected dilution for specific cDNA samples (10 ng for SbMATE transcripts and 0.01 ng for 18S RNA) were used as real-time PCR templates to quantify relative transcript levels following the conditions recommended by the manufacturer. The forward (F) and reverse (R) primers, as well as the probe sequences are F: 5′-CAG CCATTGCCCATGTTCTTT-3′, R: 5′-ACCAGCTTGCTCAGCATTATCA-3′ and Probe: 6FAM-CCCAGTACCTGATAACGC-TAMRA.

Levels of expression for endogenous 18S RNA were determined using TaqMan Ribosomal RNA Control Reagents (AppliedBiosystems, Foster City, CA). Distilled water or products of room temperature reactions without reverse transcriptase were used as negative controls. The levels of the *SbMATE* transcripts were normalized to endogenous 18S RNA and *SbMATE* expression relative to that in the Al sensitive accession, BR012, was calculated. Three technical reps were used. The experiment was repeated 2 times with similar results.

### DNA isolation and PCR amplification

Leaf tissue from three plants of each accession and leaf from individual seedlings for the segregating families were used for DNA isolation according to Saghai-Maroof and colleagues [50]. The markers CTG29 (CTG29F: HEX-ATGCAGTATCTGCAGTATCATTT; CTG29R: AATCCGTCAGGTCAGCAATC), S17 (S17F: GGCTGCCCGTCCCTTTCTCTGTCT; S17R: CCGGGGCGCTGGGCTTCCTT) and S73 (S73F: AAGCGCTGGCCCAAATGAAATGA; S73R: GAGCCAACACGGGGAGAACAAGTC) were used to determine whether Al tolerance in the tolerant sources was due to allelic variation at *Alt_SB_*. CTG29 is a sequence tagged site (STS) marker that is linked to *Alt_SB_* at 0.2 cM (estimate obtained in 2085 F_2_ individuals derived from a cross between SC283 and BR007, [7]). Upon positional cloning of *Alt_SB_* [7], another two STS markers, S17 and S73 were developed from sequences in the same bacterial artificial chromosome (BAC) that harbors *Alt_SB_*, with S17 being located at 32.1 kb from *Alt_SB_* on the same side as CTG29 whereas S73 is located at 22.4 kb from *Alt_SB_.* on the opposite side. Due to the tight physical and genetic linkage of these marker loci to *Alt_SB_*, the odds for a double recombination event in BC families of the size used in this study are extremely low, making these markers diagnostic for *Alt_SB_*.

PCR reactions with CTG29 were performed as described in Caniato *et al*. [35]. For S17 and S73, amplifications were carried out in a reaction volume of 20 µL which contained 30 ng of genomic DNA, 10× polymerase chain reaction buffer, 0.5 mM dNTP, 3 mM MgCl_2_, 4 pmol of each primer, 5% of dimethyl sulfoxide (DMSO) and 0.5 U of Taq DNA polymerase (Phoneutria, Belo Horizonte, MG). Amplification proceeded with an initial denaturation step of 95°C for 1 min followed by 30 cycles at 94°C for 1 min, 62°C for 1 min, 72°C for 1 min, and a final extension step at 72°C for 10 min. Electrophoresis was carried out in 1% (w/v) agarose gel at 100 V in 1× TAE buffer, revealing scorable polymorphisms between the parental lines.

Thirty-eight SSR markers from a sorghum SSR kit (http://sat.cirad.fr/sat/sorghum_SSR_kit/) developed within the Generation Challenge Programme (GCP), which are evenly distributed across the sorghum genome, were used for genetic diversity and population structure analyses. The fragment sizes obtained for the Deu *et al*. [34] collection were provided by the GCP and the 45 lines from the Embrapa collection were genotyped with the same SSR markers. Because differences in allele sizes for the same alleles are expected between labs, a set of 10 highly diverse sorghum lines with a wide range of allelic variation (http://sat.cirad.fr/sat/sorghum_SSR_kit/data/control_comp.html) was used as a control. DNA for these 10 lines were used for PCR amplification with the same SSR markers along with the 45 lines from the Embrapa collection to normalize differences in fragment sizes based on what was obtained under the conditions employed by GCP and Embrapa. PCR reactions were carried out as described for STS amplifications but without DMSO and using 2.5 pmol of each SSR primer. Amplification proceeded with a touchdown protocol including an initial denaturation step at 94°C for 4 min, followed by 9 cycles at 94°C for 45 s, 60°C for 1 min with a reduction rate of 0.5°C per additional cycle, 72°C for 1 min 15 s, and 24 cycles at 94°C for 45 s, 55°C for 1 min, 72°C for 1 min 15 s, and a final extension step of 5 min at 72°C. Three microliters of 200-fold diluted amplification products and 6.9 µl of Hi-Di formamide (Applied Biosystems, Foster City, CA) were mixed with 0.1 µl GS500 ROX (Applied Biosystems, Foster City, CA) internal size standard and denatured at 95°C for 5 min. The fragments were assayed on an ABI 3100 sequencer (Applied Biosystems, Foster City, CA). Fragment sizes were determined based on migration relative to the internal size standard using the GeneMapper 3.5 software. Allele sizes obtained for each control line were compared to the expected allele sizes posted on http://sat.cirad.fr/sat/sorghum_SSR_kit and a correction factor for each marker was imposed to normalize allele sizes for the GCP and Embrapa datasets so that the two panels could be genetically merged.

### Statistical analysis of Al tolerance data

One-way analysis of variance for *RNRG*_3d_, *RNRG*_5d_, *VRD* and *IRG* data at each Al activity, followed by the Scott-Knott test [51], were initially undertaken to cluster the accessions into homogeneous groups for the response variables. *RNRG*_3d_, *RNRG*_5d_ and *IRG* data obtained at {27} µM Al^3+^ were also subjected to Principal Component Analysis (PCA, [52]) based on standardized variables.

### Genetic analysis of Al tolerance control at the *Alt_SB_* locus

Simple interval mapping was undertaken when two markers flanking the *Alt_SB_* locus were available whereas single marker analysis was applied in the remaining cases. Significant associations with Al tolerance were declared at a logarithm-of-odds (LOD) equal to or higher than three.

### Gene action estimates for Al tolerance

The degree of dominance for the Al tolerance trait was estimated as the ratio between dominance (d) and additive (a) effects, d/a, where *d = Tt−[(TT+tt)/2* and *a = (TT−tt)/2. TT* denotes the *RRG* mean for the tolerant parent, *tt* is the *RRG* mean for the sensitive parent, BR007, which was common to all crosses, and *Tt* denotes the *RRG* mean for each F_1_ hybrid. Therefore, a d/a value of −1 indicates that the phenotypic mean of the F_1_ (Tt) hybrid equals that of the homozygous sensitive (tt) parent, d/a = +1 means that the F_1_ hybrid is as tolerant as the homozygous tolerant (TT) parent, and d/a = 0 indicates that Al tolerance in the F_1_ hybrid equals the average of the RRG means estimated for the two parents.

In the present study we adopted the following convention for assigning modes of gene action related to Al tolerance: recessive (d/a≤−0.7), partially recessive (−0.7<d/a<−0.3), additive (−0.3≤d/a≤+0.3), partially dominant (+0.3<d/a<+0.7) and dominant (d/a≥+0.7).

### Genetic diversity analysis

Total and per locus number of alleles and the Polymorphism Information Content (
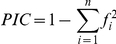
, where 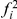 is the squared frequency of the *i*th allele), were calculated with PowerMarker version 3.25 [53].

### Analysis of population structure

A Bayesian cluster analysis as implemented in the software STRUCTURE [54], [55] was used to estimate the number of subpopulations (k) based on the SSR data set. The admixture model with correlated allele frequencies was adopted, with burn-in length 100,000 and 1,000,000 run length, with five independent runs for each k set to range from 1 to 13. It has been reported that in some instances the log probability of data may not provide a correct estimate of number of clusters [56]. Thus, we also calculated Δk as the second order change of log of probability of data, Ln(k), divided by its standard deviation [56] and the rate of change in Δk between successive k values was adopted as an auxiliary criterion to identify the most likely number of subpopulations.

### Analysis of Al tolerance with respect to subpopulations defined by STRUCTURE

The non-parametric Kruskal-Wallis test was initially used to test whether the defined subpopulations differed for the Al tolerance response variables assessed at {27} µM Al^3+^. Statistical significance for all pairwise differences among subpopulations for each variable were estimated by calculating the least significant difference (*lsd*) between subpopulations as 
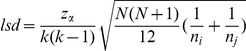
, where 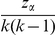 is the superior limit of the normal distribution; *n_i_* and *n_j_* are the number of individual within each subpopulation, *i* and *j*, respectively, and N is the total number of individuals.

Subsequently, a linear mixed model accounting for population structure and familial relatedness or kinship [57] was fit to the data in order to clarify a possible relationship between population structure and the distribution of the Al tolerance in sorghum. Our model was **y = Qν+Zu+e**, where **y** is a vector of phenotypic observations, **ν** is a vector of fixed effects related to population structure and **u** is a vector of random effects related to familial relatedness. **Z** is an incidence matrix of 0 s and 1 s, relating **Z** to **y**. **Q** is the population membership assignment matrix obtained with STRUCTURE. The variances for the random effects are **Var(u) = 2K** ***V*** **g** and **Var(e) = R** ***V*** **_R_**, where **K** is a 254×254 matrix based on the proportion of shared allele values [58], obtained with PowerMarker [53], **R** is a 254×254 matrix with the off-diagonal elements being zero and the diagonal elements being the reciprocal of the number of observations for which each phenotypic data point was obtained, ***V*** **g** the genetic variance, and ***V*** **_R_** the residual variance. Analyses were performed in SAS with the code available at http://www.maizegenetics.net/unified-mixed-model.

Our complete model included the subpopulations Q1, Q2, Q3, Q4 and Q6. Q5 was found to comprise basically Al sensitive genotypes and was thus excluded from the model to remove dependency. Each one of subpopulations Q1, Q2, Q3, Q4 and Q6 were then sequentially removed following model selection based on the Bayesian Information Criterion (BIC, [59]).

## Supporting Information

Figure S1 **Posterior probability of data, Ln(D), for each number of subpopulations (k).** Simulations were carried out with k ranging from 1 to 13. Ln(k) values are means of five independent runs for each k.
(DOC)

Figure S2 **Second order of change of probability of data (Δk,** [**56**] **) for different subpopulation numbers (k).**
(DOC)

Figure S3 **Membership of individual sorghum accessions to subpopulations (Q).** (A) k4Q1, guinea accessions from western Africa and guinea margaretiferum; k4Q2, durra accessions from Central eastern Africa and from Asia, bicolor and caudatum accessions from Asia; k4Q3, caudatum accessions from Africa, group of transplanted caudatum and durra accessions from Lake Chad region and lines from Embrapa collection and USA and k4Q4, kafir and guinea accessions from southern Africa and (B) k6Q1, guinea accessions from western Africa and guinea margaretiferum; k6Q2, caudatum accessions from Africa and group of transplanted caudatum and durra accessions from Lake Chad region; k6Q3, lines from Embrapa collection and US; k6Q4, kafir accessions from southern Africa; k6Q5, durra accessions from central eastern Africa and from Asia; bicolor and caudatum accessions from Asia k6Q6, guinea accessions from southern Africa and Asia. Membership coefficients for each subpopulation are shown in Table S6. Arrows indicate hierarchical subpopulation splits from k = 4 to k = 6.
(DOC)

Table S1 **Sorghum accessions evaluated in this study.** Country of origin, racial classification according to [26] performed at ICRISAT and CIRAD as found in [34] are shown followed by RNRG_3d_, RNRG_5d_, VRD and IRG evaluated at {27}µM Al^3+^ and at {39} and {60} µM Al^3+^ (except VRD). Values are means of two replications (7 plants per replication). Means followed by the same lower-case letters constitute homogeneous groups by the Scott-Knott test (P<0.05). Al tolerant accessions indicated in Figure 1 are underlined. CV: coefficient of variation.
(XLS)

Table S2 **Principal Component Analysis for** ***RNRG*** **_3d_,** ***RNRG*** **_5d_ and** ***IRG*** **.** Eingenvectors, eigenvalues and the cumulative proportion of total variance (%) explained are shown for each principal component (PC).
(DOC)

Table S3 **Marker-trait associations in backcross families.** Linkage analysis was performed with marker loci tightly linked to *Alt_SB_* and the relative root growth (RRG) phenotype.
(DOC)

Table S4 **Gene action estimates for** ***Alt_SB_*** **.** Relative root growth (%RRG) means for the Al tolerant and sensitive parents and the respective F1 hybrids are shown. Additive (a) and dominance (d) effects in addition to the degree of dominance (d/a) were estimated for each cross.
(XLS)

Table S5 **Details for SSR markers used in this study.**
(XLS)

Table S6 **Membership assignment matrix for six subpopulations.** Country of origin and racial classification according to [26] performed at ICRISAT and CIRAD as found in [34] are shown followed by each subpopulation (Q) defined with the software STRUCTURE.
(XLS)
