# Supplementary material for: The Relationship between Population Structure and Aluminum Tolerance in Cultivated Sorghum
Source: PLoS One. 2011 Jun 14;6(6):e20830. doi: 10.1371/journal.pone.0020830 (PMC3114870; doi:10.1371/journal.pone.0020830)
Supplement: Figure S1 — Posterior probability of data, Ln(D), for each number of subpopulations (k). Simulations were carried out with k ranging from 1 to 13. Ln(k) values are means of five independent runs for each k. (DOC) [file pone.0020830.s001.doc]

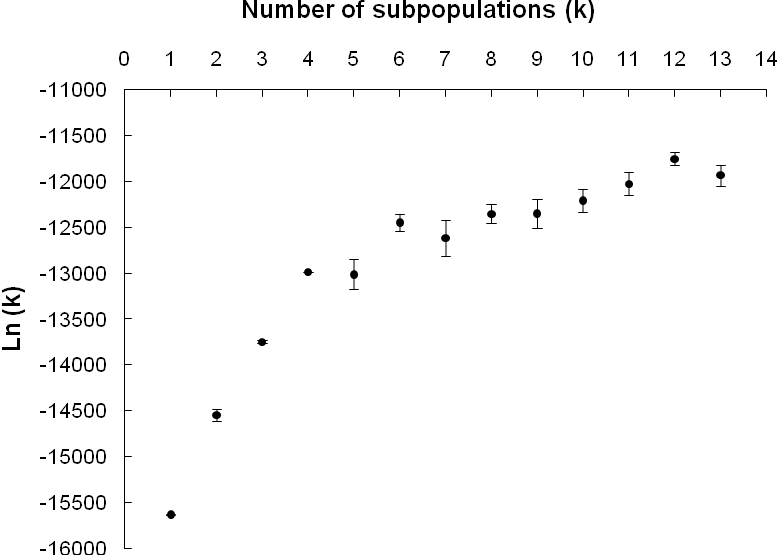


**Figure S1. Posterior probability of data, Ln(D), for each number of subpopulations (k)**. Simulations were carried out with k ranging from 1 to 13. Ln(k) values are means of five independent runs for each k.
