# Supplementary figures and images for: The Relationship between Population Structure and Aluminum Tolerance in Cultivated Sorghum
Source: PLoS One. 2011 Jun 14;6(6):e20830. doi: 10.1371/journal.pone.0020830 (PMC3114870; doi:10.1371/journal.pone.0020830)

**
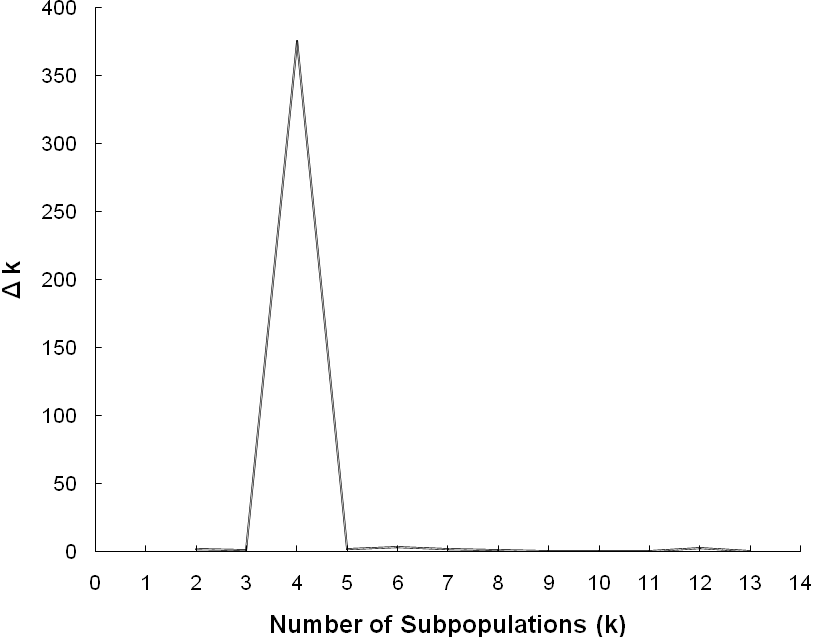
**

**Figure S2.** **Second order of change of probability of data (∆k, [57]) for different subpopulation numbers (k).**

Supplement: Figure S2 — Second order of change of probability of data (Δk, [56] ) for different subpopulation numbers (k). (DOC) [file pone.0020830.s002.doc]
