# Supplementary material for: The Relationship between Population Structure and Aluminum Tolerance in Cultivated Sorghum
Source: PLoS One. 2011 Jun 14;6(6):e20830. doi: 10.1371/journal.pone.0020830 (PMC3114870; doi:10.1371/journal.pone.0020830)
Supplement: Figure S3 — Membership of individual sorghum accessions to subpopulations (Q). (A) k4Q1, guinea accessions from western Africa and guinea margaretiferum; k4Q2, durra accessions from Central eastern Africa and from Asia, bicolor and caudatum accessions from Asia; k4Q3, caudatum accessions from Africa, group of transplanted caudatum and durra accessions from Lake Chad region and lines from Embrapa collection and USA and k4Q4, kafir and guinea accessions from southern Africa and (B) k6Q1, guinea accessions from western Africa and guinea margaretiferum; k6Q2, caudatum accessions from Africa and group of transplanted caudatum and durra accessions from Lake Chad region; k6Q3, lines from Embrapa collection and US; k6Q4, kafir accessions from southern Africa; k6Q5, durra accessions from central eastern Africa and from Asia; bicolor and caudatum accessions from Asia k6Q6, guinea accessions from southern Africa and Asia. Membership coefficients for each subpopulation are shown in Table S6. Arrows indicate hierarchical subpopulation splits from k = 4 to k = 6. (DOC) [file pone.0020830.s003.doc]

**A**

**B**

**Figure S3**. **Membership of individual sorghum accessions to subpopulations (Q).** (A) k4Q1, guinea accessions from western Africa and guinea margaretiferum; k4Q2, durra accessions from Central eastern Africa and from Asia, bicolor and caudatum accessions from Asia; k4Q3, caudatum accessions from Africa, group of transplanted caudatum and durra accessions from Lake Chad region and lines from Embrapa collection and USA and k4Q4, kafir and guinea accessions from southern Africa and (B) k6Q1, guinea accessions from western Africa and guinea margaretiferum; k6Q2, caudatum accessions from Africa and group of transplanted caudatum and durra accessions from Lake Chad region; k6Q3, lines from Embrapa collection and US; k6Q4, kafir accessions from southern Africa; k6Q5, durra accessions from central eastern Africa and from Asia; bicolor and caudatum accessions from Asia k6Q6, guinea accessions from southern Africa and Asia. Membership coefficients for each subpopulation are shown in Table S6. Arrows indicate hierarchical subpopulation splits from k=4 to k=6.
