# Supplementary material for: The Relationship between Population Structure and Aluminum Tolerance in Cultivated Sorghum
Source: PLoS One. 2011 Jun 14;6(6):e20830. doi: 10.1371/journal.pone.0020830 (PMC3114870; doi:10.1371/journal.pone.0020830)
Supplement: Table S2 — Principal Component Analysis for RNRG 3d, RNRG 5d and IRG . Eingenvectors, eigenvalues and the cumulative proportion of total variance (%) explained are shown for each principal component (PC). (DOC) [file pone.0020830.s005.doc]

**Table S2. Principal Component Analysis for *RNRG*_3d_, *RNRG*_5d_ and *IRG*.** Eingenvectors, eigenvalues and the cumulative proportion of total variance (%) explained are shown for each principal component (PC).

| **Variables** | **PC1** | **PC2** |
| --- | --- | --- |
| *RNRG*_3d_ | 0.58 | -0.57 |
| *RNRG*_5d_ | 0.61 | -0.17 |
| *IRG* | 0.54 | 0.80 |
| Eigenvalue | 2.60 | 0.36 |
| Cumulative Variance | 86.6 | 98.7 |
