# Supplementary material for: The Relationship between Population Structure and Aluminum Tolerance in Cultivated Sorghum
Source: PLoS One. 2011 Jun 14;6(6):e20830. doi: 10.1371/journal.pone.0020830 (PMC3114870; doi:10.1371/journal.pone.0020830)
Supplement: Table S3 — Marker-trait associations in backcross families. Linkage analysis was performed with marker loci tightly linked to AltSB and the relative root growth (RRG) phenotype. (DOC) [file pone.0020830.s006.doc]

**Table S3. Marker-trait associations in backcross families. Linkage analysis was performed with marker loci tightly linked to *Alt_SB_* and the relative root growth (RRG) phenotype.**

| **Accession** | **Markers** | **N** | **LOD** |
| --- | --- | --- | --- |
| IS14351 | S17-S73 | 112 | 24.53 |
| IS21519 | S17-S73 | 102 | 4.70 |
| IS21849 | S17-S73 | 94 | 7.90 |
| IS23142 | CTG29 | 97 | 0.13 |
| IS26457 | S73 | 72 | 0.57 |
| IS23645 | S17-S73 | 62 | 4.22 |
| IS26554 | S17-S73 | 102 | 4.63 |
| IS29691 | CTG29 | 91 | 0.009 |

Significant association were declared at LOD>3.0

N: number of individuals
